# Online conversations on perceived stigma among pregnant individuals who use cannabis

**DOI:** 10.1016/j.dadr.2025.100352

**Published:** 2025-06-18

**Authors:** Lindy K. Howe, Lauren Micalizzi, Mary Ellen Fernandez Goyette, Elizabeth R. Aston, Rachel L. Gunn

**Affiliations:** a Center for Alcohol and Addiction Studies, Department of Behavioral and Social Sciences, Brown University School of Public Health, Providence, RI, USA; b Department of Psychiatry and Human Behavior, Alpert Medical School of Brown University, Providence, RI, USA

**Keywords:** Cannabis, Pregnancy, Forum, Stigma, Perinatal, Qualitative

## Abstract

Perinatal cannabis use (PCU) is a controversial topic, as it is associated with negative neonatal and maternal outcomes. However, PCU persists, often reported in the context of perceived symptom management, and those who engage in PCU can face significant stigma. Such stigma can deter individuals from discussing their cannabis use with support persons, potentially exacerbating negative health outcomes for the parent and child. The current study explores how PCU stigma manifests and is navigated in online communities, focusing on discussions within an online space for individuals who use cannabis during pregnancy. First, a random sample of 10 threads per month from June 2020 to May 2021 were extracted from a cannabis-focused forum on a pregnancy and parenting website. Next, analyses involved a two-cycle coding process (i.e., topical followed by conceptual) to identify key themes surrounding stigma in the context of PCU. Three primary themes were identified: 1) experiencing stigma (e.g., familial and societal sources of judgment, emotional responses such as guilt and frustration); 2) contradictions in substance stigma, highlighting the contrast between societal attitudes and expectations towards cannabis versus alcohol or tobacco use during pregnancy; and 3) challenging stigma, in which participants actively provide support, share personal experiences, and offer evidence to counteract stigma. Online communities may play a critical role in combating stigma, offering a platform for connection, support, and education. Results emphasize that a nonjudgmental approach to information dissemination may be more effective. Understanding stigma is essential for developing effective interventions and reducing the harms of PCU.

## Highlights

• Perinatal cannabis use is increasing, despite known risks to parent and child health.
• Stigma around perinatal cannabis use affects access to care and support networks.
• Qualitative analysis of online forum with two-cycle coding to identify stigma themes.
• Forum members report stigma from society and peers, reporting guilt and frustration.
• Contradictions emerge between societal views on cannabis vs. alcohol/tobacco use.

## 1 Introduction

Perinatal cannabis use (PCU) is a public health concern, with past month prenatal cannabis use rates reaching 7 % in the 2017 National Survey on Drug Use and Health (NSDUH) (Volkow et al., 2019), and is linked to negative health outcomes for both mother and baby, including preterm birth, low birthweight, and increased neonatal care admissions (Grant et al., 2018, Prewitt et al., 2023). Up to 65 % of individuals that discontinue cannabis use in pregnancy resume use after delivery (Eitel et al., 2024) often reporting low risk perception and noted medicinal benefits to mental and physical health (Boerner et al., 2024, Smith et al., 2024). In one sample of the 5.5 % of individuals that returned to cannabis use postpartum, 47 % reported cannabis while breastfeeding (Coy et al., 2021). Delta-9-tetrahydrocannabinol (THC), the primary psychoactive component in the cannabis plant, crosses the placenta and accumulates in breast milk, with fetal exposure linked to adverse health outcomes such as motor delays (Bertrand et al., 2018, Coy et al., 2021). Despite these risks, PCU is prevalent, and rates are increasing (Gesterling and Bradford, 2022, Barbosa-Leiker et al., 2020, Brown et al., 2017), underscoring the need for a deeper understanding of the attitudes and perspectives surrounding this issue. Notably, many individuals report experiencing benefits from cannabis use during pregnancy, often using it for self-treatment to manage pregnancy symptoms or chronic pain (Eitel et al., 2024, Vanstone et al., 2021). Negative perception of individuals based on their perinatal cannabis use (i.e, stigma) surrounding PCU may deter pregnant individuals from sharing their positive experiences with cannabis as symptom management or disclosing their cannabis use to healthcare providers (Bright et al., 2022), worsening health risks (Greene et al., 2023, Stengel, 2014). However, little is known about how individuals experience stigma and understanding these experiences is essential for developing better support and intervention strategies.

### 1.1 Negative effects of stigma

Stigma refers to the negative perception or disapproval of individuals or groups based on characteristics or behaviors deemed socially undesirable or deviant (Link and Phelan, 2001, Yang et al., 2017). Theories of stigma highlight that individuals with stigmatized traits are seen as "less than" or inferior, leading to social exclusion and discrimination (Goffman, 1963; 2009), and underscores how societal structures (e.g., institutions) reinforce negative attitudes and inequalities toward stigmatized groups (Link and Phelan, 2001). Stigma can have significant negative consequences and individuals are often viewed as irresponsible or morally failing (Kulesza et al., 2013, Yang et al., 2017), which can result in discrimination and hinder access to support or treatment (Kulesza et al., 2013, Yang et al., 2017). This may be particularly salient for pregnant people, who experience social pressures and expectations of sacrifice in relation to their developing child (Kahalon et al., 2021, van Mulken et al., 2016).

Stigma in healthcare often manifests through negative biases and discriminatory attitudes held by healthcare providers, which can lead to treatment disparities and a lack of trust between patients and providers (Riffel and Chen, 2020). For individuals with substance use disorders or who use cannabis, this stigma is amplified, as individuals are often perceived as lacking self-control or moral character, rather than as individuals experiencing a medical condition that requires support and treatment (King et al., 2024, van Boekel et al., 2013). These biases can deter individuals from seeking care, adhering to treatment plans, or disclosing important health information, ultimately affecting their health outcomes and access to quality care (Zwick et al., 2020). Understanding stigma helps identify negative effects on clinical populations and informs strategies to address and reduce stigma, ultimately improving healthcare outcomes.

### 1.2 PCU related stigma

Perceived stigma is particularly significant for pregnant and postpartum individuals, as health warnings discourage cannabis use. However, many use cannabis during pregnancy to manage symptoms like nausea, pain, or anxiety (Gunn et al., 2024, Bayrampour et al., 2019, Vanstone et al., 2022). Individuals seeking relief through cannabis often face judgment (Greene et al., 2023, Raifman et al., 2024). While healthcare providers are advised to discourage cannabis use during pregnancy due to potential risks (Passey et al., 2014, Young-Wolff et al., 2020), this can unintentionally reinforce stigma by not offering non-judgmental support for those using cannabis (van Boekel et al., 2013) and potentially experiencing a cannabis use disorder. This lack of support undermines efforts to promote maternal and infant health (Schiff et al., 2022, Weber et al., 2021). Fear of judgment can prevent individuals from disclosing cannabis use to healthcare providers, limiting early intervention and potentially worsening health outcomes (Hammarlund et al., 2018, Kulesza et al., 2013, Luoma et al., 2007, Daniels et al., 2023). Stigma also deepens health disparities, particularly for marginalized populations, where limited access to care increases the risk of adverse pregnancy outcomes (Philippopoulos et al., 2024, Stringer and Baker, 2018).

As a result, many individuals adjust their behavior in healthcare settings (Biancarelli et al., 2019) or turn to alternative sources for information, such as friends, family, and online communities (Jarlenski et al., 2016, O’Dowd et al., 2025, Oni et al., 2022). However, pregnant individuals often face stigma about cannabis use from their families as well (Barbosa-Leiker et al., 2020). Regardless of the source, this stigma can lead to shame, isolation, and distress (Kirkbride et al., 2024, Ahad et al., 2023), worsening maternal mental health (Birtel et al., 2017), and contributing to negative pregnancy outcomes like preterm birth and low birth weight (Voit et al., 2022). Additionally, perceived stigma can drive individuals to withdraw from support networks, internalize shame, and limit access to care and information (Hammarlund et al., 2018, Milan and Varescon, 2024), reducing the protective social support that helps prevent negative maternal and infant outcomes (Birtel et al., 2017, Chou et al., 2018).

Online forums provide a space where individuals may feel less vulnerable to judgment (Lebron et al., 2022, Liu et al., 2019, Taneja et al., 2023). The perinatal population frequently turns to these platforms for information and support (Taneja et al., 2023). Anonymity encourages candid conversations (Johnson, 2015), offering a unique opportunity to explore lived experiences of those facing stigma during pregnancy. However, the information shared can sometimes be low-quality or harmful (Ellis and Roberts, 2020). Despite this, analyzing data from online forums remains a valuable method for gaining insights into cannabis use during pregnancy (Ellis and Roberts, 2020, Gunn et al., 2024, Lebron et al., 2022, Micalizzi et al., 2024, Moore et al., 2016). For example, a recent study explored how pregnant and postpartum individuals in a supportive cannabis use forum perceive the developmental effects of PCU on their children, finding that posters discussed both positive and negative impacts on child development, sought information from non-medical sources, and shared harm-reduction strategies to mitigate potential risks (Micalizzi et al., 2024). Additionally, Lebron et al. (2022) found that online forums provide a space for mothers who use cannabis to share concerns, particularly about regulations and experiences across different U.S. regions. However, none of these studies have specifically focused on stigma related to PCU. Further research is needed to understand how stigma discussed in these forums may reinforce continued use and contribute to the harmful outcomes associated with PCU.

### 1.3 The current study

PCU-related stigma can paradoxically contribute to continued use, yet much remains unknown about how this manifests, persists, and affects individuals' coping mechanisms and support-seeking behavior. To improve prenatal and postpartum care and reduce stigma’s unintended consequences, it is crucial to gather detailed evidence on how individuals experience and respond to stigma related to PCU. This study uses qualitative data from a cannabis-focused forum on a pregnancy and parenting website to gain insights into PCU-related stigma.

## 2 Method

### 2.1 Qualitative data preparation

The analysis focused on a forum that is ideal for the research focus as it is commonly used by expectant parents seeking information and support and reflects perspectives of individuals actively engaged in pregnancy-related decision-making. All threads posted between June 2020 and May 2021 (time of extraction) were extracted. Using a Python script with a randomization function, a random sample of 10 forum threads per month was selected for analysis, resulting in a total of 120 threads. Threads were included in the current analysis if they specifically addressed “cannabis use during pregnancy” or “breastfeeding”, as determined via review conducted by least two independent coders. Threads that discussed cannabis use outside the context of pregnancy or lactation were excluded. Seven threads did not include reference to “breastfeeding” or “cannabis” specifically during pregnancy, resulting in a final dataset of 113 threads for coding and analysis.

### 2.2 Data analysis

A deductive coding framework was employed for initial data analysis (Bingham, 2023), drawing on existing literature to inform the coding process, which was adjusted as necessary. All threads underwent two cycles of coding: topical (Cycle 1), then conceptual (Cycle 2).

#### 2.2.1 Cycle 1 coding

In Cycle 1 coding, relevant quotes were reviewed by two coders using applied thematic analysis and each post was evaluated to identify key topics using an open coding process (Linneberg and Korsgaard, 2019). As the coding process advanced, codes were continuously refined, and related codes were grouped to form themes. Coders gathered weekly to achieve consensus and address coding discrepancies, engaging in intensive discussions, coder adjudication, and basic consensus-building to resolve discrepancies and indicate agreement (Harry et al., 2005, Brinkmann and Kvale, 2015, Colditz et al., 2018). Finalized codes were organized in NVivo (QSR International Pty Ltd., 2020) to streamline synthesis and categorization. Codes pertaining to “stigma” were then exported for Cycle 2 coding.

#### 2.2.2 Cycle 2 coding

All codes pertaining to PCU-related stigma underwent a second round of coding, focusing on categorization, integration, synthesis, and conceptualization of the topics. Cycle 2 criteria for inclusion of quotes required explicit reference to an experience of judgment, stigma, or negative views specifically related to cannabis use during pregnancy. Quotes that did not meet this criterion were excluded. The data were then independently summarized by two coders to identify key themes. The coders achieved thematic consensus and selected representative quotes to illustrate each theme (see Micalizzi et al., 2024 for details).

### 2.3 Transparency and openness

The data used in this study did not require registration for access. Forum users were anonymous, identified only by usernames, and no personal information was collected. As such, the study did not qualify as research involving "human subjects" and did not require institutional review board approval. To ensure anonymity, usernames were omitted, the data will not be publicly shared, and quotes were minimally altered to prevent identity disclosure, as retaining the original text could reveal contributors via searching for their quotes using a search engine. This approach is common in the field and maintains meaning while safeguarding privacy (Colditz et al., 2018; Zimmer, 2010).

## 3 Results

Three themes emerged: 1) experiencing stigma, 2) perceived contradictions in substance stigma, and 3) challenging stigma, each with several subthemes. A summary of themes with quote examples is presented in Table 1.

**Table 1:** Study sub-themes and example quotations.

Sub-theme	Description	Example Quotation
Theme 1: Experiencing Stigma		
1.1 Familial Sources	Posters reflect on the ways in which family is the source of PCU stigma.	My grandpa has always been judging my weed use
1.2 Societal beliefs and expectations	Societal attitudes relative to PCU stigma.	Society makes it seems like it’s horrible but that is an outdated way of thinking….
1.3 Responses to stigma	Posters reflect on emotions relative to PCU stigma: 1) guilt/shame and 2) frustration/anger	
1.3.1 Guilt/Shame		I felt bad because many people were bashing me for smoking…
1.3.2 Frustration/Anger		[General public] hides their smoking from their kids…it really annoys me
Theme 2: Contradictions in substance stigma		
2.1 Alcohol	Posters reference differences in PCU stigma and alcohol stigma.	I was shamed for smoking…shortly after that I was offered shots of liquor
2.2 Cigarettes	Posters reference differences in PCU stigma and cigarette stigma.	The stigma on weed and not on cigarettes makes me really beyond mad
Theme 3: Challenging Stigma		
3.1 Providing evidence against	Posters shared personal successes with PCU despite stigma	I smoked my whole pregnancy and I want to show other moms that may be worried about smoking when pregnant…it wont harm your baby…my baby is thriving at 6 months old
3.2 Seeking understanding	Posters asked for support in the context of PCU stigma	…I’ve seen lots of moms smoke during their entire pregnancy then stop smoking…I don’t understand. What is the difference?
3.3 Providing support	Posters offered support and validation in the context of PCU stigma	Remember, [weed] is medicine, use it as such and don't listen to the shamers…Good luck mommas

### 3.1 Experiencing stigma

Group members reflected on stigma from personal encounters and societal attitudes, sharing the emotional impact of these experiences.

#### 3.1.1 Familial sources

Forum members shared situations in which they experienced stigma from those around them, such as their significant other/partner, parents and family, and in-laws. For some, their partner (i.e., significant other – SO) voiced judgements placed on the poster due to their beliefs surrounding PCU. One poster expressed “*it's…my significant other making me feel guilty…how he looks at me when I mention smoking…”*. Families also expressed explicit judgement, with a member commenting “*My grandpa has always been judging my weed use”*. Another poster shared their attempts at discussing PCU with a loved one, explaining, “*[Poster’s father referring to reducing use early in pregnancy]…you obviously don’t need it if you went this long without it…he goes on some lecture about needing to grow up not smoke weed cuz I have a baby now and the vices need to stop. the “you need to grow up” part really got under my skin…so I ignored it”.* The same poster echoed sentiments from other posters by explaining such beliefs were unfair judgements, stating “[Poster’s father] *won’t hold my baby or change the diaper cause he doesn’t do that….but says I need to grow up and stop using weed?!”.* Several posts identified their in-laws (i.e., someone who is a relative because of marriage) as one source of stigma, with one poster sharing, “*my in-laws freaked out when they found out I’m smoking and breastfeeding”* and another forum member expressed that it was difficult to explain their situation to in-laws, stating, “*everyone views smoking pot in different ways so I…understand not…being able to explain your pot use to your in laws”.* While many reported judgements from others in their life, one commenter expressed the lack of judgement from people on the forum, “*moms here will not bother judging me”.*

#### 3.1.2 Societal beliefs and expectations

Several forum members remarked on general negative societal beliefs related to PCU, saying “*society makes it seems like it’s horrible but that is an outdated way of thinking. You and your baby will be okay”.* A poster responded to a photo of a seemingly healthy baby, stating, “*The [referencing baby photo] is how [the baby in the photo] feels about the negative people that talked bad about smoking while pregnant…[Baby is saying]‘look at me, healthy and beautiful”.*

Some posters noted society's positive view of cannabis use, but highlighted the stigma around prenatal and parental cannabis use, with one remarking, “*I know other people who smoke and I feel like they only do it because they think they look cool. Even still, they hide their smoking from their kids”.* Some individuals talked about negative experiences with other baby-specific forums, “*baby center is rude as [expletive]”.*

Diverging from other posters, a member voiced their views on cannabis use in pregnancy related to overall beliefs and expectations of being a parent, “*I prefer my baby to be born without needing to withdrawal from THC. While I’m a stoner, my baby shouldn’t start out as one… All I have to say is, it is not just about you anymore”.*

#### 3.1.3 Responses to stigma

Posters also expressed strong emotional reactions, including guilt, shame, frustration, or anger.

##### 3.1.3.1 Guilt/shame

Those who experienced stigma directly stated they had guilty reactions to perceived stigma from multiple people, with one poster commenting “*I felt bad because many people were bashing me for smoking…so I smoked less, but even then I just had to sometimes…and it helped a lot”.* Another poster reflected about guilt after judgement from a loved one, stating “*I feel sort of guilty and worried that I am not in the right frame of mind”.* For some, shame related to PCU was brought to the forum, with a member directly asking “*Am I a really bad person for smoking?…I just smoke 2 or 3 hits at night when I go to sleep”?*

##### 3.1.3.2 Frustration/Anger

In the context of stigma, all posters expressing frustration or anger cited societal beliefs or personal experiences. One poster explicitly stated their anger, saying “*I am beyond mad. I’m so annoyed!!!”.* Several other commenters expressed their reaction as annoyance, including posts reacting to other people’s hypocritical beliefs. One individual explained, “*[the general public] hides their smoking from their kids, calling weed something else like pineapple and it really annoys me*”. One expressed frustration that stigma was still present in a state where cannabis is legalized, commenting “*I’m in California, making it even more annoying”.* Overall, individuals were disheartened by PCU-related stigma, with one poster stating, “*Really got under my skin”.* Some came to the forum to specifically share about recent stigmatizing experiences. A poster included a story of a recent stigmatizing situation, ending with “*My evening was really frustrating and i wanted to vent it all out to get it off my chest at how annoyed i am. I feel like that was very hypocritical for him to say and I hate hypocritical people”.*

### 3.2 Contradictions in substance stigma

A key theme was the stigma specifically directed at cannabis, unlike alcohol or cigarettes, which posters found contradictory.

#### 3.2.1 Alcohol

All posts highlighting the contradiction between alcohol and cannabis stigma noted that alcohol was less stigmatized. One member stated they felt more stigmatized for PCU than those who drank alcohol during pregnancy, expressing, “*[Other people] get drunk right in front of the kids, and get belligerent, which I think is way worse”.* Some were even offered alcohol while pregnant, explaining they were “*I was shamed for smoking…shortly after that I was offered shots of liquor”.* Another poster similarly commented, “*Why is it okay to drink in front of kids, but smoking in front of them is not okay!? I feel like parenting and smoking at the same time is much better than drinking and parenting”.*

#### 3.2.2 Cigarettes

All posts in this subtheme noted that cigarettes are harmful, yet those around them demonize cannabis, and not tobacco/cigarettes. One individual commented, “*My grandma used to smoke cigarettes right in front of me as a kid and no one thought twice”.* Beyond this, several posters noted that cannabis is specifically stigmatized over other drugs, with one individual stating, *“the stigma on weed and not on cigarettes makes me really beyond mad”.* An individual experienced PCU-related stigma while the person judging was smoking cigarettes, noting, “*[father] was smoking so many cigarettes around me and talking about how I shouldn’t be even smoking weed when pregnant!”.* Finally, one poster noted a hypocritical tone to a comment made about their ability to stop PCU, stating that their “*[Dad] was proud of me when I quit smoking [weed] and…he never could have given cigarettes even if his life depended on it”.* Thus, despite their father’s inability to quit, the poster was still expected to.

### 3.3 Challenging stigma

Comments challenged stigmatizing views by requesting or offering support and sharing personal or external evidence.

#### 3.3.1 Providing evidence against stigma

Some challenged stigmatizing beliefs by sharing personal success stories despite cannabis use. One individual commented, “*Despite the fact that I graduated from college, held down a job since I was 15 years old, I have lived on my own since I was in early 20s, have a career, own my own house…some people will still be judgmental regardless of success or how much proof you have to show that cannabis does not negatively impact you”.* Several posts included evidence that PCU is okay because their child is healthy despite PCU. One individual stated, “*They induced me at before 40 weeks due to my age. My baby is perfect even though I smoked throughout pregnancy”.* Another poster explained, “*I smoked my whole pregnancy and I want to show other moms that may be worried about smoking when pregnant…it wont harm your baby…my baby is thriving at 6 months old”.*

Another reference included research/articles that they found to help manage perceived stigma related to PCU, saying, “*for anyone looking for more research/articles…i found these helpful for dealing with judgement/anxiety…let me know if you find them helpful”.*

##### 3.3.1.1 Seeking understanding

Some sought support and understanding, with one poster asking readers to “*Please keep negative judgments to yourself”.* Another individual sought understanding regarding stigma-related guilt, asking, “*I have a serious question…no judgment. Why do people feel guilty about smoking since your baby is here and healthy? I’ve seen lots of moms smoke during their entire pregnancy then stop smoking because of guilt once the baby is born and I don’t understand. What is the difference?*”. Several members sought to discuss cannabis stigma, posting, “*Why are we still demising and is there a taboo on weed*….*I can’t wrap my head around why”.*

##### 3.3.1.2 Providing validation

Members provided support to those posting about experienced stigma, such as insisting they don’t listen to harmful judgement, and expressing validation. For example, one poster wrote, “*Remember, [weed] is medicine, use it as such and don't listen to the shamers…Good luck mommas”.* Another member supported a previous commenter in their stigmatizing experience, stating, “*Don’t let him get to you!…that first joint after birth will be magical”.*

Further, several posts sought to combat stigma by expressing positive appreciation and validation to moms, with one poster sharing, “*It's been good to hear positive comments and have discussions without judgment. Thank you to this community for support! The people on What to Expect Are Great!”.* Validation was common, with members explaining “*It is difficult because people have different views on smoking pot so I totally understand not wanting or feeling able to explain your situation”,* and “*You are completely fine! I think it’s normal”.*

## 4 Discussion

The current study evaluated posts from an online pregnancy forum geared towards pregnant women that use cannabis. The forum provided a space for individuals to discuss their experiences and seek support without judgement, and results suggest stigmatizing experiences lead to frustration, seeking support, and placing emphasis on personal experience (i.e., rather than evidence-based information).

While PCU-related stigma is often highlighted as a significant issue in healthcare settings (King et al., 2024, Daniels et al., 2023, van Boekel et al., 2013), results indicate that individuals also experience stigma in their personal lives, particularly from loved ones and within close social circles. Findings suggest that members of the forum experienced stigma from familial sources, such as family members, friends, and the broader societal judgment of cannabis use. Anger and frustration were commonly expressed in response to these stigmatizing experiences, highlighting the emotional toll stigma can take on individuals during pregnancy, which can be harmful to the mother or developing fetus (Dunkel Schetter and Tanner, 2012, Jimènez-Barragan et al., 2024). This highlights that while PCU can be harmful, the barriers to seeking support is also a significant concern, especially during pregnancy, a time when individuals may already be facing discrimination and stigma related to general behaviors. Systems should focus on creating spaces where individuals feel safe seeking help, ensuring that they can receive the support they need without fear of judgment. By doing so, individuals may be better positioned in situations where they can receive informed, evidence-based information.

Forum participants also shared experiences of feeling unfairly stigmatized, particularly in comparison to the other substances such as alcohol and tobacco. Alcohol and tobacco are widely used in the U.S. (Hasin et al., 2007, Passey et al., 2014, Holt et al., 2012, Shmulewitz and Hasin, 2019) and are associated with health risks both in the general population (Lu et al., 2023) and during pregnancy (Passey et al., 2014, Shmulewitz and Hasin, 2019). Posters also seemed to recognize that individuals who are not currently pregnant may perpetuate stigma or hold views on PCU despite lacking lived experience, highlighting the unique discrimination individuals face specifically in relation to pregnancy and reproduction. Forum members highlighted inconsistency in how stigma is applied to cannabis use versus alcohol and tobacco use, despite all substances being linked to known pregnancy-related risks (Passey et al., 2014). As individuals’ health decisions are influenced strongly by societal attitudes (Kilmer et al., 2007), it is important to recognize how societal attitudes contribute to harms and risks during pregnancy. Inherent to some of the posts related to “contradictions in drug stigma” was a context of familial discord (i.e, “[father] was smoking so many cigarettes around me and talking about how I shouldn’t be even smoking weed when pregnant!). It should be noted that the source of distress relative to PCU stigma may come from multiple sources (i.e., contradictions and familial sources), which may perpetuate the heightened emotions and response to stigma. Further, perceived contradictions may stem from the inconsistent messaging surrounding cannabis use (McKenzie et al., 2022). While alcohol and cigarette risks are consistently communicated in public health and clinical settings (Golechha, 2016, Pettigrew et al., 2023, Polańska et al., 2015), cannabis messaging remains ambiguous and source dependent. This lack of clarity may be compounded by ambiguous guidance from healthcare professionals (Taneja et al., 2023, Vanstone et al., 2022). To address this gap, it is crucial to develop clear, evidence-based public health campaigns that consistently communicate the risks of PCU.

Additionally, forum members offered support through personal anecdotes (e.g., their child is healthy despite exposure to cannabis), and highlighted how these experiences contradict current guidelines for PCU. Previous work analyzing qualitative reports from focus groups yielded similar results, with participants reporting risk perceptions of PCU being shaped strongly by anecdotal stories from friends and family (McKenzie et al., 2022, Micalizzi et al., 2024) rather than evidence-based sources. Posters shared personal stories of cannabis use during pregnancy with no perceived negative effects, potentially reinforcing the view that it isn’t harmful. While these anecdotes offer support, they may challenge public health guidelines and spread misinformation. Anecdotal experiences may be particularly influential for those who experience positive medicinal benefits from cannabis (Boerner et al., 2024). Subsequent research on pregnant individuals using cannabis should assess recreational and medical motives, as differences in these motives may require distinct preventative and harm-reduction approaches. Providers may also benefit from proactively creating an environment that fosters open discussions about the benefits versus risks of cannabis use. In short, healthcare providers should value lived experiences but emphasize evidence-based guidance to prevent harm.

### 4.1 Limitations

The findings should be viewed considering several limitations. First, the anonymous nature of the forum prevented assessment of demographic characteristics, limiting generalizability, especially regarding stigmatizing experiences common in marginalized communities (Nidey et al., 2022, Young-Wolff et al., 2021). Second, the forum's explicit focus on cannabis use may introduce selection bias, making findings less applicable to those without a positive bias toward cannabis. Third, due to feasibility constraints, only a random sample of 120 posts from a single year were analyzed. This limited sample may not fully capture the diversity of discussions on the forum, and it is possible that the nature of conversations may vary over time. Additionally, results should be considered within the context of the timeline relative to the COVID-19 pandemic as posts were from May 2020-June2021. While this was not within the scope of the current study, and information on exact extraction dates is not included, it would be fruitful for future research to explore whether and how these conversations changed as pandemic-related restrictions eased. Lastly, the study lacked data on specific events prompting posts, limiting the ability to interpret content beyond what was explicitly shared and potentially overlooking important contextual factors that could influence the discussion.

### 4.2 Conclusion and future directions

In conclusion, this study underscores that part of the harm associated with PCU-related stigma, which creates additional barriers to seeking support and care. While the risks of PCU are well-documented and dissemination is crucial, stigma further exacerbates these challenges, hindering access to necessary resources. Future research should focus on how stigma impacts maternal and infant health outcomes and examine ways to disseminate evidence-based in a non-stigmatizing way. Further, more research is needed on prevention efforts and improving communication between providers and patients regarding PCU. Ultimately, fostering nonjudgmental spaces in perinatal care will help combat stigma, improve support, and may mitigate the harmful effects of PCU.

## CRediT authorship contribution statement

**Lauren Micalizzi:** Conceptualization, Data curation, Formal analysis, Investigation, Methodology, Project administration, Resources, Supervision, Writing – original draft, Writing – review & editing. **Howe Lindy K:** Conceptualization, Formal analysis, Methodology, Software, Writing – original draft, Writing – review & editing. **Elizabeth R. Aston:** Conceptualization, Data curation, Formal analysis, Investigation, Methodology, Project administration, Resources, Software, Supervision, Writing – original draft, Writing – review & editing. **Mary Ellen Fernandez Goyette:** Conceptualization, Project administration, Writing – review & editing. **Rachel L. Gunn:** Conceptualization, Data curation, Investigation, Methodology, Project administration, Supervision, Writing – original draft, Writing – review & editing.

## Declaration of Competing Interest

The authors declare that they have no known competing financial interests or personal relationships that could have appeared to influence the work reported in this paper
